# How dangerousness evolves after court-ordered compulsory psychiatric admission: explorative prospective observational cohort study

**DOI:** 10.1192/bjo.2019.21

**Published:** 2019-04-04

**Authors:** Mark H. de Jong, André I. Wierdsma, Antonius W. B. van Baars, Arthur R. Van Gool, Cornelis L. Mulder

**Affiliations:** Psychiatrist, Yulius Mental Health, Dordrecht, The Netherlands; Assistant Professor of Social Psychiatry, Epidemiological and Social Psychiatric Research Institute, Department of Psychiatry, Erasmus University Medical Centre, Rotterdam, The Netherlands; Psychiatrist, Department of Psychiatry, Bravis Hospital, Roosendaal, The Netherlands; Psychiatrist, Yulius Mental Health, Dordrecht, The Netherlands; Psychiatrist, Professor of Public Mental Health, Epidemiological and Social Psychiatric Research Institute, Department of Psychiatry, Erasmus University Medical Centre, Rotterdam, The Netherlands

**Keywords:** Court-ordered admission, dangerousness, in-patient treatment

## Abstract

**Background:**

Compulsory admission is commonly regarded as necessary and justified for patients whose psychiatric condition represents a severe danger to themselves and others. However, while studies on compulsory admissions have reported on various clinical and social outcomes, little research has focused specifically on dangerousness, which in many countries is the core reason for compulsory admission.

**Aims:**

To study changes in dangerousness over time in adult psychiatric patients admitted by compulsory court order, and to relate these changes to these patients' demographic and clinical characteristics.

**Method:**

In this explorative prospective observational cohort study of adult psychiatric patients admitted by compulsory court order, demographic and clinical data were collected at baseline. At baseline and at 6 and 12 month follow-up, dangerousness was assessed using the Dangerousness Inventory, an instrument based on the eight types of dangerousness towards self or others specified in Dutch legislation on compulsory admissions. We used descriptive statistics and logistic regression to analyse the data.

**Results:**

We included 174 participants with a court-ordered compulsory admission. At baseline, the most common dangerousness criterion was inability to cope in society. Any type of severe or very severe dangerousness decreased from 86.2% at baseline to 36.2% at 6 months and to 28.7% at 12 months. Being homeless at baseline was the only variable which was significantly associated with persistently high levels of dangerousness.

**Conclusions:**

Dangerousness decreased in about two-thirds of the patients after court-ordered compulsory admission. It persisted, however, in a substantial minority (approximately one-third).

**Declaration of interest:**

None.

Although compulsory admissions are commonly seen as necessary and justified for patients whose psychiatric condition is a severe danger to themselves and others, they also represent a serious breach of personal autonomy.^1^ Judicial procedures and reported numbers of compulsory admissions differ considerably between countries:^2^ although the core criterion for compulsory emergency admission in the USA and many European countries is mental illness resulting in danger to self or others, further criteria vary.^3,4^ While certain countries apply two criteria – both need for treatment and dangerousness – three European states apply one criterion for compulsory admission: the need for treatment caused by a mental illness.^4^

Studies of compulsory admission and its follow-up after discharge have reported on the following: clinical and social outcomes and the motivation for treatment,^5,6^ perceived coercion,^7,8^ patients' views on whether the admission was right,^9^ and family perspective.^10^ However, there has been very little research on the core reason for compulsory admission, which is dangerousness to self and others. In a study published in 1988, the associations between clinically assessed criteria of dangerousness (danger to self, danger to others and grave disability) and diagnosis and psychopathology at admission were studied.^11^ Dangerousness was associated with major mental disorder and with the severity of most symptom types. Danger to self had the fewest associations with indicators of mental disorders.^11^ Despite studies in the field of forensic psychiatry, we found no studies in general psychiatry that investigated dangerousness in the context of court-ordered compulsory admission. To date, it is thus an open question in general psychiatry whether and to what extent compulsory admission contributes to reducing dangerousness to self and others. Neither is it known whether subgroups of patients can be identified whose levels of dangerousness to themselves and others are persistently high despite compulsory admission. Conceivably, dangerousness is greater and more persistent in patients whose symptoms are more severe at the start of compulsory admission, whose illness insight is poorer, and whose treatment engagement is poor.^12^ Other factors – such as gender, age, symptom severity, diagnosis, substance misuse, illness insight and compliance with treatment – may also be relevant to predicting the level and persistence of dangerousness.

As care may be improved by identifying these subgroups of patients and then devoting extra attention to them, we wished to study the development of dangerousness to self and others over a 12 month period in adult psychiatric patients who had been admitted compulsorily to a general psychiatric hospital. We also wished to identify any associations between the development of dangerousness and these patients' baseline demographic and clinical characteristics.

## Method

### Study design and setting

This 12 month explorative prospective observational cohort study involved adult psychiatric patients who had been admitted compulsorily to a psychiatric hospital in Rotterdam Rijnmond, an urban area in The Netherlands with 1.2 million inhabitants. Patients in The Netherlands can be compulsorily admitted if they have a mental disorder resulting in danger to self and others, if there is no alternative way of averting the danger, and if they do not actively consent to the admission. Under The Netherlands' Exceptional Admissions to Psychiatric Hospitals Act (Dutch abbreviation: BOPZ) there are two main procedures for compulsory psychiatric admission, one in which emergency compulsory admission is sanctioned by the local mayor, and one in which court-ordered admission is sanctioned by a judge. If a patient is acutely dangerous to himself or others, the emergency procedure is applied, which requires admission within 24 h. The hospital stay of a patient under an emergency procedure can be extended by a court-ordered admission. A court-ordered admission can also be applied when a patient is not in need of acute admission but represents a danger to himself or others. Assessment by an independent psychiatrist is mandatory for both types of compulsory admission. After examining the patient, the independent psychiatrist completes a specific mental health act form, describing the diagnosis and the dangerousness criteria applicable, including the most important dangerousness criterion. After examination by an independent psychiatrist, a judge decides whether the court-ordered admission is justified.

This study focused on patients whose admission had been ordered by a court, whether or not they had had a previous emergency compulsory admission. Over a period of 18 months, patients were recruited from the psychiatric services in Rotterdam Rijnmond, which consist of three general psychiatric hospitals, and the psychiatric department of a university medical centre. The follow-up period was 12 months. Assessments took place at baseline, after 6 months and after 12 months.

The authors assert that all procedures contributing to this work comply with the ethical standards of the relevant national and institutional committees on human experimentation and with the Helsinki Declaration of 1975, as revised in 2008. All procedures involving patients were approved by the medical ethics committee at Erasmus University Medical Centre with approval number MEC-2004077.

### Participants

Adult patients (>18 years old) became eligible as soon as a clinician had requested a court-ordered admission. Written informed consent was obtained from all patients. To ensure that hospital stay durations at baseline were comparable, informed consent, study inclusion and baseline assessment were scheduled no more than 4 weeks after the clinician had started the procedure for a court-ordered admission. Exclusion criteria were organic psychiatric disease (e.g. Alzheimer's disease) and rejection by the court of the index request for compulsory admission.

### Baseline assessment

After informed consent had been given, we collected the following information from the electronic patient files: demographic characteristics (age, ethnicity, marital status, education level, homelessness) and psychiatric history (previous voluntary and/or compulsory admissions). We also collected the most important dangerousness criterion stated by the independent psychiatrist. All participants were interviewed using the Composite International Diagnostic Interview^13^ for the assessment of mental disorders according to the DSM-IV.^14^ Psychiatric symptom severity was assessed using the Brief Psychiatric Rating Scale;^15^ insight was assessed using the Birchwood self-report Insight Scale^16^ and the Schedule of Assessment of Insight-Expanded version (staff-rated);^17^ and compliance and service engagement were assessed using the Service Engagement Scale.^18^

### Dangerousness Inventory

As a tool to assess severity of dangerousness to self and others in general psychiatric patients, we developed a Dangerousness Inventory (DI). Danger to self was assessed on the basis of four items derived from the legal text in the Dutch BOPZ act: the risk that

- the individual will die by suicide or inflict severe self-harm;
- the individual will fail to cope in society;
- the individual will seriously neglect himself or herself;
- through his or her behaviour, the individual will elicit the aggression of others.

Danger to others was also assessed on the basis of four items derived from the legal text: the risk that

- the individual will commit murder or seriously harm others;
- the individual will be a burden on the mental health of others;
- the individual will neglect those for whose care he or she is responsible;
- the individual will endanger the overall safety of people and goods.

Each item was rated on a five-point scale ranging from no danger (score = 0) through slight danger (score = 1), moderate danger (score = 2) and, severe danger (score = 3), to very severe danger (score = 4). To explain the meaning of the different ratings and to train the assessors, we used concise descriptions of the respective scores per item as well as case vignettes.

The DI and the other instruments were rated by independent interviewers (medical and psychological students who had received training in scoring the instruments) on the basis not only of patient interviews and observation, but also of interviewing admission ward psychiatrists to obtain information on the type and severity of dangerousness. Interrater reliability of the DI was checked in a subsample of 45 patients. The kappa values of most items were in the 0.61 to 0.80 range, indicating substantial interrater agreement.

### Statistical analysis

At baseline, descriptive statistics were used to describe patients' characteristics and the type and severity of dangerousness. As a cut-off score for dangerousness, we used severe to very severe danger (DI score ≥3). At baseline, 6 months and 12 months, we calculated the proportion of all participants with DI scores ≥3. This was done for all eight DI items combined, for the four DI items of danger to self combined, and for the four DI items of danger to others combined. This enabled us to describe the evolution of dangerousness in terms of the proportion of patients who were persistently dangerous. To indicate severity of danger, we also calculated the number of DI items ≥3 (indicating high or very high level of dangerousness) at baseline per individual participant. Additionally, we identified a danger-to-self subgroup and danger-to-others subgroup. These were based on dangerousness at baseline and also on the most important type of danger indicated in the request for court-ordered admission by the independent consulting psychiatrist. All further analyses were performed for these subgroups separately. To calculate the associations between patients' baseline characteristics and change in dangerousness, we performed explorative logistic regression analysis. We chose the 6 months follow-up time point for the main logistic regression analysis, because the initial court ordered admission was sanctioned for a period of 6 months. We also performed additional analyses for the 12 months follow-up time point. Variable selection was based on a stepwise procedure suggested by Hosmer & Lemeshow, with *P* = 0.20 and 0.05 levels of entry and removal.^19^ Model fit for the resulting model was assessed using the receiver-operating characteristics curve and Hosmer–Lemeshow chi-squared analysis. To evaluate differences in outcomes related to the choice of cut-off values, sensitivity analyses were performed. In this exploratory approach, no adjustments for multiple testing were made. The data were analysed using the SPSS 24.0 statistical package.

## Results

### Participants

**Fig. 1:**
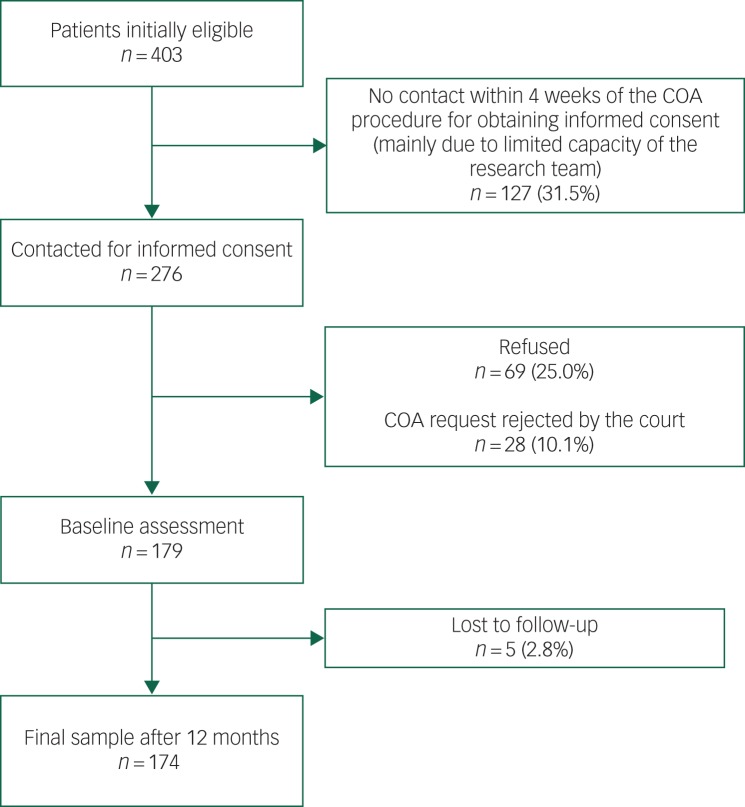
Flow-chart of the participant selection process.

We included 174 of the patients who had initially been eligible. The numbers and reasons for exclusion are shown in Fig. 1.

COA, court-ordered admission.

Baseline demographic and clinical characteristics are summarised in Table 1. There were high proportions of the following patients: single males, patients diagnosed with schizophrenia, those with an earlier compulsory admission, those with severe symptoms and those with limited illness insight. All are typical of patients admitted by court order.

**Table 1:** Baseline characteristics of patients admitted by court order (*n* = 174)

	*n* (%)	Mean (SD)
Age (years)		35.1 (14.0)
Gender (male)	117 (67.2)	
Ethnicity		
Dutch	72 (41.4)	
Surinamese/Antillean	36 (20.7)	
Turkish/Moroccan	30 (17.2)	
Other	36 (20.7)	
Marital status		
Single	126 (72.4)	
Married	18 (10.3)	
Divorced/widowed	30 (17.2)	
Low educational level	88 (50.6)	
Homeless	22 (12.6)	
Diagnosis		
Schizophrenia	118 (67.8)	
Other psychotic disorder	19 (10.9)	
Affective disorder	33 (19.0)	
Comorbid substance misuse (except nicotine)	66 (37.9)	
Untreated psychosis >1 year	31 (17.8)	
Previously admitted compulsorily	87 (50.0)	
BPRS^a^		58.0 (12.2)
BIS^b^		4.9 (3.8)
SAI-E^c^		12.0 (6.2)

a. BPRS = Brief Psychiatric Rating Scale.

b. BIS = Birchwood Insight Scale.

c. SAI-E = Schedule of Assessment of Insight-Expanded version.

### Dangerousness at baseline

Table 2 shows the numbers and proportions (%) of all participants with a score ≥3 per DI item at baseline. Inability to cope in society was by far the most common dangerousness criterion. The proportions of serious self-neglect, eliciting aggression from others, murder or serious harm to others, and being a burden on the mental health of others were all considerably lower, lying in the 20–30% range.

**Table 2:** Dangerousness criteria^a^ at baseline (number and percentage of all participants)

Criterion	(Very) severe danger^b^	
*Danger to self*	*n*	%
1. Suicide/severe self-harm	16	9.2
2. Inability to cope in society	99	56.9
3. Serious self-neglect	52	29.9
4. Eliciting aggression from others	50	28.7
*Danger to others*	*n*	*%*
5. Murder or serious harm	45	25.9
6. Being a burden on the mental health of others	38	21.8
7. Neglecting dependent others	10	5.7
8. Endangering overall safety	24	13.8

a. Patients can be dangerous in more than one way at the same time.

b. DI score ≥3.

At baseline assessment, the number of DI items scored ≥3 per participant ranged from zero to six. The largest proportion of participants (73.5%) showed one to three DI items ≥3. It is notable that 13.8% of the participants scored no DI items ≥3, which means that, according to the DI, they showed neither severe nor very severe danger at baseline. This can be explained by the fact that the baseline assessment was carried out as soon as possible after the court-ordered admission. When the baseline assessment took place, participants had thus been in hospital for a short while.

### Evolution of dangerousness

**Fig. 2:**
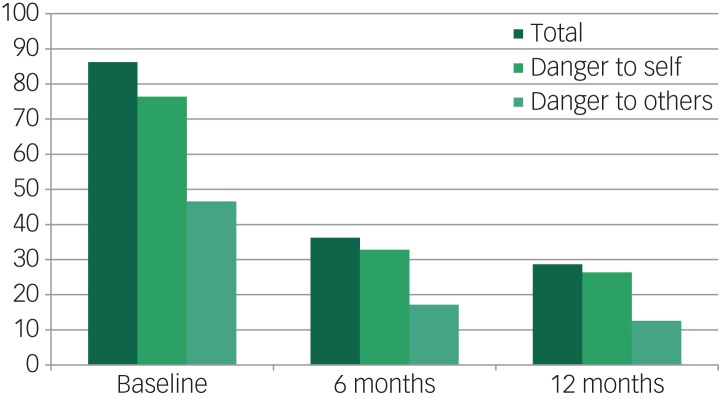
Evolution of dangerousness (≥1 DI item ≥3), total, danger to self and danger to others (percentage of all participants).

At baseline, 86.2% of the participants showed dangerousness (defined as ≥1 DI item ≥3). Separate examinations of the scores for the DI items for danger to self and danger to others showed that the proportion of scores ≥3 for any item of danger to self was 76.4%, while the proportion of scores ≥3 for any item of danger to others was 46.6%. At 6 months follow-up, these proportions had fallen to 36.2% (all items), 32.8% (danger to self) and 17.2% (danger to others). At 12 months follow-up, the proportions were again slightly lower (Fig. 2). In other words, whereas dangerousness (≥1 DI item ≥3) disappeared from a majority of the participants within 6 months of admission, it persisted in a substantial minority (28.7%) even after 12 months. There was also a smaller minority of 19 participants (10.9%) in whom dangerousness was absent (no score ≥3 at all) at 6 months but in whom it recurred at 12 months.

### Predictive factors associated with the evolution of dangerousness

In the danger-to-self subgroup, the persistence of danger to self (DI ≥ 3) at 6 months follow-up was associated with age, being homeless, previous compulsory admission, and high dangerousness at baseline (univariate predictors). Being homeless was the only predictor that showed significance in the multivariate analysis (B = 1.368, SE = 0.615, *P* = 0.026). In the danger-to-others subgroup, persistence of danger to others (DI ≥ 3) at 6 months follow-up was associated with ethnicity and being homeless (univariate predictors). In the multivariate analysis, however, ethnicity was not statistically significant, unlike being homeless (B = 2.466, SE = 1.272, *P* = 0.053).

In the additional analyses at 12 months follow-up, being homeless at baseline did not remain a statistically significant predictor for persistence of danger (DI ≥ 3) in either the danger-to-self subgroup or the danger-to-others subgroup. This was no surprise, because the group with persistent dangerousness at 12 months follow-up was smaller than that at 6 months follow-up and partly consisted of different patients.

These results were not affected in sensitivity analyses by a different cut-off score (DI ≥ 2) or by a slightly different allocation to the respective subgroups.

## Discussion

### Main outcome

This explorative prospective observational study explored the evolution of dangerousness after court-ordered compulsory admission. At baseline, the most common dangerousness criterion was inability to cope in society. During the 12 month observation period, levels of dangerousness levels decreased considerably in a majority of participants. In our view, this is a meaningful and clinically relevant observation. However, it also important to note that dangerousness persisted in a substantial minority of patients. In both the danger-to-self and the danger-to-others subgroups, persistent dangerousness at 6 months follow-up was associated with homelessness at baseline (before compulsory admission).

### Strengths and limitations

This is the first study in general psychiatry to examine the evolution of dangerousness after compulsory admission of psychiatric patients. As it was a prospective study that included patients with a minimum of exclusion criteria, its results reflect the outcomes of a patient group that is representative of a psychiatric hospital population in a Dutch urban setting.

The first limitation is that the observational nature of the study makes it impossible to draw conclusions with regard to the causal relationships among compulsory admission, the evolution of dangerousness and patient characteristics. Second, we had no information on the duration of the hospital stay. Thus, we were not able to calculate the association between persistence of dangerousness and length of stay. We might nonetheless expect duration to be longer in patients in whom high levels of dangerousness persisted. Third, we included 174 (43%) of 403 originally eligible patients. As we did not have data for the patients who refused to participate or who were lost to contact, the findings of this study might be subject to some selection bias. Finally, the DI used in the present study had not yet been validated and, as the DI is based on Dutch legislation, its generalisability to other countries is unknown. Most countries, however, do apply danger to self and others as dangerousness criteria, as is the case in The Netherlands.

Future studies on dangerousness of compulsorily admitted patients should try to optimise the participation rate of eligible patients, e.g. by offering an incentive for participation. It would also be interesting to assess a wider set of clinical variables in order to obtain more detailed knowledge of factors associated with persistence or decrease of dangerousness. Our exploratory analysis suggests that patients' characteristics and psychopathology are not strong predictors of dangerousness. However, several clinical characteristics were not assessed, including impulsivity, urgency, self-control and conscientiousness, which could be addressed in future studies.

Moreover, future studies could validate the DI against other scales, such as the MacArthur Violence Risk Assessment,^20^ by assessing specific types of danger in relation to clinical and demographic variables. Similar studies in other parts of the world are needed to improve the generalisability of our findings.

### Clinical implications

By providing insight into the evolution of dangerousness in psychiatric patients who have been admitted compulsorily, this study demonstrates that these hospital admissions are associated with a decrease in dangerous behaviour of these patients. This result may be interpreted as providing some support for the statement above that autonomy should ultimately be breached in patients whose psychiatric condition is a severe danger to themselves and others. A subgroup of patients – those who are homeless – may deserve extra clinical attention and more research on how to reduce their seemingly persistent high level of dangerousness.
